# Observation of the analgesic effect of superficial or deep anterior serratus plane block on patients undergoing thoracoscopic lobectomy

**DOI:** 10.1097/MD.0000000000024352

**Published:** 2021-01-22

**Authors:** Lan Qiu, Xiaoxuan Bu, Jiang Shen, Min Li, Linyi Yang, Qingrong Xu, Yongjun Chen, Jianping Yang

**Affiliations:** a Department of Anesthesiology, Intensive Care Medicine and Pain Medicine, the First Affiliated Hospital of Soochow University, Suzhou; b Department of Anesthesiology; c Department of Cardiology, The Third Affiliated Hospital of Soochow University, Changzhou, Jiangsu, China.

**Keywords:** deep anterior serratus plane block, superficial anterior serratus plane block, thoracoscopic surgery, ultrasound guidance

## Abstract

The effectiveness of anterior serratus plane block in postoperative analgesia of thoracic surgery is beginning to emerge. Currently, there are 2 methods of anterior serratus plane block: deep serratus plane block (DSPB) and superficial serratus plane block (SSPB). In clinical practice, there is no an unified view regarding the advantages and disadvantages between 2 methods. This study aimed to observe and compare the analgesic effects of 2 methods on patients undergoing thoracoscopic lobectomy, in order to provide some suggestions for anesthesiologists when they choose anterior serratus plane block to perform postoperative analgesia for patients.

Patients were randomly divided into 3 groups (21 patients/group):

1. general anesthesia group (P group);
2. combined general anesthesia and SSPB group (S group), and
3. combined general anesthesia and DSPB group (D group).

The patients in groups S and D received 0.4 ml/kg of 0.375% ropivacaine for ultrasound-guided block after surgery. Postoperatively, flurbiprofen was used for rescue analgesia.

Visual analog scale (VAS) pain scores were recorded at 6 hours, 12 hours, and 24 hours after surgery, and rescue analgesia, post-operative nausea, and vomiting were reported within 24 hours after surgery. At 6 hours, 12 hours, and 24 hours, the VAS scores and the rescue analgesia rates in groups S and D were significantly lower than those in group *P* (all *P* < .001). With prolonging time, the VAS in group D was significantly increased by 0.11 per hour as compared with that of group *P* (*P* < .0001); VAS in group D was significantly increased by 0.12 per hour as compared with that of group S (*P* < .0001).

Ultrasound-guided anterior serratus plane block can provide adequate analgesia for patients undergoing thoracoscopy lobectomy. SSPB can significantly improve VAS scores as compared to DSPB at 24 hours.

## 1 Introduction

Video-assisted thoracoscopic surgery (VATS) is a less invasive treatment method. Compared with thoracotomy, patients have less postoperative pain and better oxygenation.^[1]^ However, patients may still suffer moderate to severe pain, especially within the first 24 hours after VATS.^[2,3]^ Postoperative pain affects patient's productive cough and expectoration, which can lead to complications, such as pulmonary infections and the delayed recovery.^[4,5]^ Adequate perioperative analgesia can reduce the adverse effects of thoracic surgery on lung function.^[6]^ Various analgesic methods have been tried to relieve pain after VATS in the clinics, but it is still difficult to achieve complete relief.^[7]^

Serratus anterior plane block (SAPB) has been confirmed to reduce opioid consumption and increase patient's satisfaction with postoperative analgesia in thoracotomy, breast surgery, rib fracture surgery, and liver surgery, etc.^[8–13]^ However, it is still controversial whether superficial serratus plane block (SSPB) or deep serratus plane block (DSPB) is more effective in the SAPB technique.^[11,14]^ Because of this, clinical anesthesiologists often confuse in choosing either SSPB or DSPB when considering the use of serratus anterior plane block (SAPB) to benefit postoperative analgesia in thoracoscopic patients.

Therefore, we conducted this study to determine which type of SAPB technology can be more effective in alleviating postoperative pain in patients undergoing VATS. We recruited patients with pulmonary nodules undergoing thoracoscopy and randomly divided them into 3 groups: DSPB, SSPB, and control group. Based on VAS scores obtained from continuous observation of these patients at multiple time points, we hypothesized that SAPB could be effective in alleviating postoperative pain in patients undergoing VATS and that the 2 SAPB methods have different effects.

## 2 Materials and methods

### 2.1 General information

After being approved by the Ethics Committee of the Third Affiliated Hospital of Soochow University and obtaining written informed consent from all the participating patients, we assessed a total of 70 patients for eligibility with exclusion criteria as follows: patients with a history of local anesthetic allergy, abnormal coagulation, infection in the puncture site, pretreatment with opioids and nonsteroidal anti-inflammatory drugs. Finally, we enrolled 66 qualified adult patients with pulmonary nodules undergoing elective thoracoscopic surgery from May 2018 to April 2019. They aged from 32 to 78 years and had ASAs I to III. Patients were randomly allocated to 3 groups using a computer-generated list of random numbers and the sealed envelopes. On the day of surgery, a nurse anesthetist allocated the participants by opening the next sealed opaque envelope, numbered them 1 to 66. The qualified patients were randomly divided into 3 groups of 21 patients each:

1. general anesthesia group (P group),
2. combined general anesthesia and SSPB group (S group), and
3. combined general anesthesia and DSPB group (D group).

### 2.2 Routine anesthesia method

All the patients were fasting for 8 hours and drinking for 2 hours without any preoperative medication. The multi-functional monitor electrocardiogram, heart rate, blood pressure, pulse oximetry, end-expiratory CO_2_ partial pressure (PETCO_2_), and anesthesia depth (BIS) were performed. Anesthesia was induced with midazolam, propofol, sufentanil, and cis-atracurium. When the patient lost consciousness, double-lumen tracheal intubation was performed. Anesthesia was induced by pumping with propofol, remifentanil. Sufentanil was added intermittently during surgery and 0.1 μg /kg sufentanil was added ten minutes before skin suture. Neuromuscular blockade was maintained with cis-atracurium. The tidal volume during the single-lung ventilation was set to 5 to 6 ml/kg and the breathing frequency was adjusted to maintain PETCO_2_ at 35 to 45 mm Hg and the depth of anesthesia maintains the BIS values between 40 and 60. Prophylactic antiemetics were administered at the discretion of the attending anesthetist.

### 2.3 Needle injection method

After surgery, the patients in groups S and D remained in the lateral decubitus position. We used ultrasound (Navi S, Wisonic, Shenzhen, Guangdong, China) with a linear ultrasound transducer (6 12 MHz). After sterilization was performed again, the musculature of the thoracic wall, including the latissimus dorsi (superficial and posterior and teres major (superior), was identified sonographically. Finally, the serratus anterior (deep and inferior) and the probe were placed parallel to and between the 4th and 5th ribs in the mid-axillary region.

**Figure 1:**
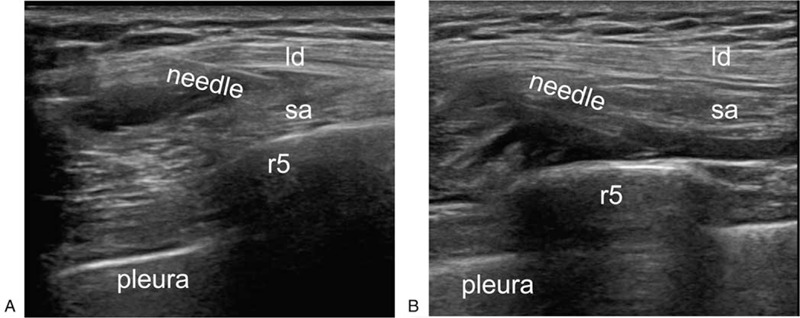
SSPB was performed by an in-plane technique using nerve block needle (a); DSPB was performed by an in-plane technique using nerve block needle (b). ld = latissimus dorsi, r5 = rib5, sa = serratus anterior.

Using a 21G × 100 mm nerve block needle (Pajunk UniPlex NanoLine, Pajunk Medical Produkte GmbH, Germany) and an in-plane technique, we injected 5 ml of saline between the latissimus dorsi and the serratus anterior muscle of patients in Group S. After confirming negative aspiration, 0.4 ml/kg of 0.375% ropivacaine was injected (Fig. 1a). The plane is hydro-dissected until the eye sign, a fusiform, hypoechoic spread of local anesthetic were seen.

The block procedure for patients in Group D was the same as that described above. However, they were administered towards the plane deep in the serratus anterior muscle. Under real time US, 5 ml of normal saline was injected to check and open the potential space underneath the serratus anterior muscle, and then 0.4 ml/kg of 0.375% ropivacaine was injected (Fig. 1b). All the blocks were performed by a single practitioner.

### 2.4 Patient-controlled intravenous analgesia pump formula

After completion of surgery, the patients were transferred to the postanesthesia care unit and attached with a PCA device immediately. The PCA regimen was consisted of administration of a total of 100 ml of 1 μg/ml sufentanil in saline. The PCA device was programmed to provide 2 μg boluses on demand, with a lockout period of 10 minutes and a background infusion at the rate of 2 ml/hour. In the case that the pain relief was inadequate after a top-up dose (VAS score >4), a rescue analgesics was provided via intravenous administration of 50 mg flurbiprofen by the acute pain service (APS) team, and it could be repeated if necessary. The maximum daily dose of flurbiprofen should not exceed 200 mg.

### 2.5 Observation and recording indicators

All the data were recorded by an investigator who was blinded to the group allocation and was not involved or present during surgery. Postoperative pain was recorded for each patient using the VAS and scored at the 6th, 12th, and 24th hours after the surgery by the APS team. The score criteria were set as follows: 0 -10, 0 points, no pain; 1 to 3 points, mild pain, without affecting sleep; 4 to 6 points, moderate pain; 7 to 9 points, severe pain, unable to fall asleep or awake during sleep; and 10 points, severe pain. The incidences of postoperative rescue analgesia, nausea, and vomiting were recorded for each patient.

### 2.6 Statistical analysis and data processing

Independent sample *t* tests or one-way ANOVA was used to compare continuous variables that are normally distributed. Non-normally distributed continuous variables were compared using the Mann–Whitney *U* test and the Kruskal–Wallis H test.

A generalized additive mixed model (GAMM) was applied to examine changes in VAS grouping over time for different anesthesia methods. GAMM eliminates the effect of individual differences on the results of repeated measurements by introducing random effects and improving statistical efficiency. All the analyses were performed using R, version 3.4.3 (http://www.R-project.org). *P* < .05 was considered statistically significant, and all the statistical tests were two-sided.

## 3 Results

**Figure 2:**
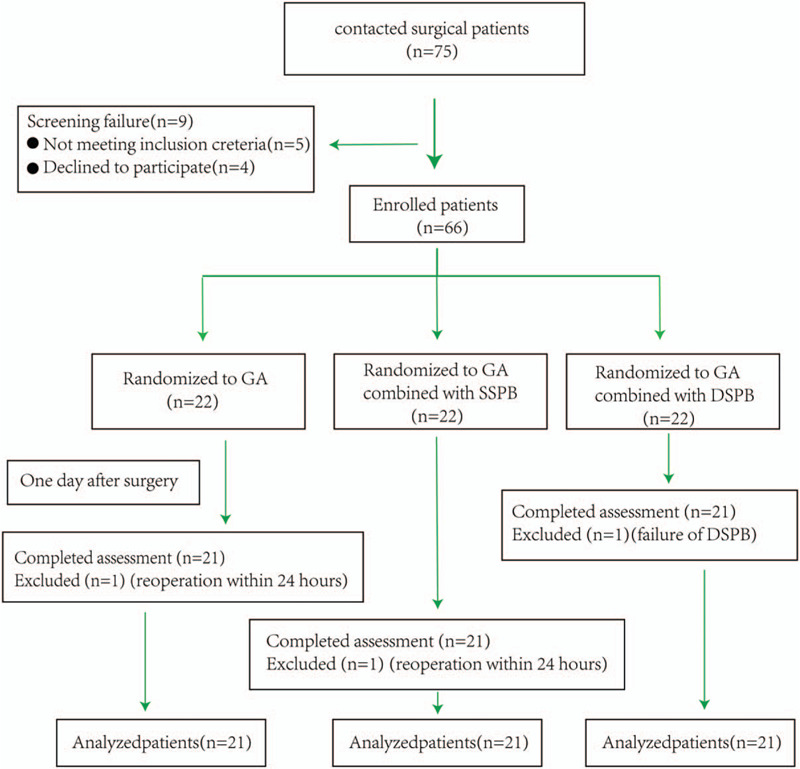
CONSORT chart outlining patient selection in the trial. DSPB = deep anterior serratus plane block, GA = general anesthesia, SSPB = superficial anterior serratus plane block.

We initially included a total of 66 patients, 2 of whom were excluded due to reoperation within 24 hours, and 1 was withdrawn due to block failure. Finally, a total of 63 qualified patients, including 33 males and 30 females with an average age of 63.5 ± 8.4 years ranged 32 to 78 years, completed the project (Fig. 2). They were divided according to anesthesia methods into P group (21 cases), S group (21 cases), and D group (21 cases). There were no statistically significant differences in general information, medication dosage and anesthesia time among patients among 3 groups (all *P* > .05), as shown in Table 1.

**Table 1:** General information about patients in 3 anesthesia groups, medication doses, and anesthesia times.

Blocking method	P Group	S Group	D Group	*P* value
N	21	21	21	
Age (year)	64.9 ± 8.3	62.7 ± 8.1	63.0 ± 8.9	.660
Sex				.466
Female	12 (57.1%)	10 (47.6%)	8 (38.1%)	
Male	9 (42.9%)	11 (52.4%)	13 (61.9%)	
Body height (cm)	164.2 ± 8.4	163.3 ± 7.1	166.2 ± 6.4	.427
Body weight (kg)	60.8 ± 8.7	62.4 ± 11.7	64.6 ± 7.4	.422
Body index (kg/m^2^)	22.5 ± 2.4	23.3 ± 3.4	23.4 ± 2.4	.520
Systolic pressure (mmHg)	126.7 ± 9.2	127.8 ± 8.9	127.5 ± 11.8	.931
High blood pressure disease	4 (19.0%)	5 (23.8%)	2 (9.5%)	.463
Diabetes	4 (19.0%)	3 (14.3%)	1 (4.8%)	.367
Hyperlipidemia	1 (4.8%)	1 (4.8%)	0 (0.0%)	.597
Maximum diameter of ulmonary nodule di (cm)	2.6 ± 0.9	2.7 ± 1.1	2.3 ± 1.0	.320
Sufentanil (μg)	48.6 ± 7.0	49.9 ± 9.4	51.7 ± 5.9	.422
Remifentanil (μg)	370.4 ± 160.3	384.5 ± 145.8	362.6 ± 109.9	.877
Cisatracurium (mg)	44.2 ± 10.0	44.4 ± 7.3	42.3 ± 5.9	.645
Propofol (mg)	632.3 ± 267.2	655.8 ± 243.0	619.4 ± 183.2	.877
Anesthesia time (min)	120.9 ± 49.9	122.1 ± 36.4	111.7 ± 29.4	.647

Table 2 shows the comparison in VAS, rescue analgesia, and PONV incidence in patients among 3 different anesthesia groups and at different time points. We found that at 6 hours, 12 hours, and 24 hours postoperation, the VASs in groups S and D were significantly lower than those in group P (all *P* < .001). The VAS in group D was higher than that in group S at 24 hours (*P* < .001), but no significant difference in VAS was seen between 6 hours and 12 hours time points (*P* = .262 and .178).

**Table 2:** VAS, rescue analgesia and PONV incidence at different time points in the 3 anesthesia groups.

	P Group	S Group	D Group	*P* value
N	21	21	21	
VAS (6 h)	5.0 (4.0–6.0)	1.0 (1.0–2.0)^#^	1.0 (0.0–1.0)^#^	<.001
VAS (12 h)	5.0 (4.0–6.0)	1.0 (0.0–1.0)^#^	1.0 (1.0–2.0)^#^	<.001
VAS (24 h)	5.0 (3.0–6.0)	1.0 (0.0–1.0)^#^	3.0 (1.0–4.0)^#^^,^^∗^	<.001
Rescue analgesia	17 (81.0%)	4 (19.0%)^#^	5 (23.8%)^#^	<.001
Nausea and vomit	5 (23.8%)	7 (33.3%)	4 (19.0%)	.556

The percentages of rescue analgesia of groups S and D were significantly lower than that (81.0%) of group P (both *P* < .001). The percentage (19.0%) of rescue analgesia of group S was lower than that (23.8%) of group D, but the difference was not statistically significant (*P* = .707); there was no significant difference in the incidence of PONV among 3 groups as well (*P* = .556).

**Figure 3:**
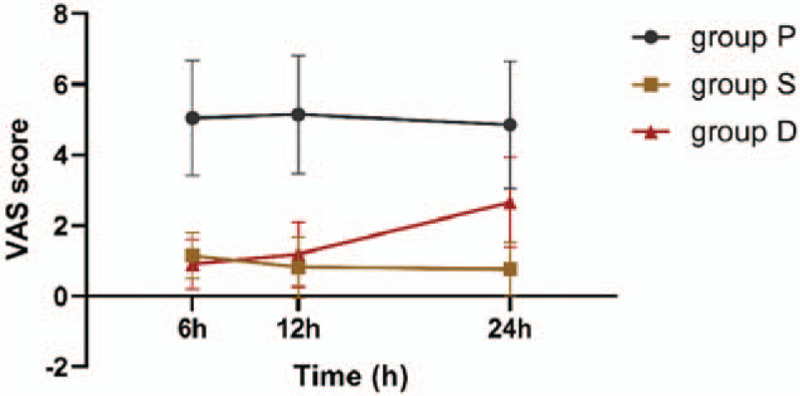
VAS score curves of 3 anesthesia methods over time (6 hours, 12 hours, and 24 hours). VAS = visual analog scale.

We further plotted the VSA curve over time for the 3 anesthesia modes (Fig. 3). With prolonging time, VASs in Groups P and S showed gradually but not significantly declining trends with regression coefficients of −0.01 and −0.02 and *P* values of .557 and .126, respectively, but VAS in group D showed a significantly increasing trend with regression coefficient of 0.10 (*P* < .0001). Further comparisons between groups revealed that the VAS in group S was reduced by 0.01 per hour as compared with that of group P, but the change was not statistically significant (*P* = .797); the VAS in group D was significantly increased by 0.11 per hour as compared with that of group P (*P* < .0001); VAS in group D was significantly increased by 0.12 per hour as compared with that of group S (*P* < .0001).

## 4 Discussion

In this study, we found that the serratus anterior plane block (SAPB), either SSPB or DSPB, can provide excellent perioperative analgesia for patients undergoing thoracoscopy lobectomy. In general, the pain is caused by the damage to the ribs, muscles, and soft tissues at the chest incision.^[15]^ Therefore, epidural block, paravertebral block, erector spinae plane block, and anterior serratus plane block have become the essential components of multimodal analgesia during perioperative thoracoscopy surgery.^[16–18]^ Due to the side effects of PVB, and the fact that it is challenging to perform, PVB may not be the best choice in postoperative pain management. Bleeding in a loose space limits the value of PVB in patients being treated with anticoagulants or antiplatelet agents.^[19]^ Besides, it is not an easy technique to place and fix the catheter for continuous analgesia in PVB. SAPB is a new regional nerve block technique for the analgesia of the lateral chest wall during and after surgery. The serratus anterior muscle originates on the surface of the first 8 ribs and attaches to the medial border of the scapula, and the posterior aspect of the latissimus dorsi. The intercostal nerves pierce it. The anterior branches of intercostal nerves traverse the deep and anterior planes of the serratus anterior muscle in transit to the subcutaneous tissue.^[20]^ Therefore, SSPB can enable patients to obtain adequate pain relief along T2 to T9. In the present study, we confirmed that patients had better pain control after SAPB. This observation is consistent with those made in previous similar studies. Additionally, compared with other analgesic techniques in thoracic surgery, such as intercostal and paravertebral block (PVB) or epidural analgesia (EA), SAPB is easier to learn and safer, since the serratus muscle is a superficial and easily identified.

More importantly, by combination with the baseline VAS and VAS change trend over time, SSPB has obvious advantages: hourly VAS in group receiving DSPB was significantly increased as compared with that of group receiving SSPB. SSPB is an ultrasound-guided technique for injecting local anesthetics into the fascial space between the anterior serratus and latissimus dorsi muscles to block the peripheral nerves, that is, the lateral cutaneous, and thus, provides excellent chest wall analgesia. It is worth noting that recent studies have reported that SSPB can significantly help patients with pain associated with chest tumor surgery,^[21,22]^ although similar effects of DSPB have also been reported in several previous studies.^[23,24]^ In the present study, we observed that the analgesic effect of SSPB was more stable and lasting-longer than that of DSPB. The serratus anterior muscle (SAM) is innervated solely by the long thoracic nerve (LTN) (C5–C7). Contraction of SAM after VATS may irritate the injured intercostal muscles and increase their tension, thereby increasing postoperative pain. In addition, movements of the shoulder or arm, and even contraction of the SAM caused by inspiration could aggravate postoperative pain after VATS, regardless of any injury to the SAM by the trocar during VATS. Since LTN lie on the surface of the anterior serratus muscle, SSPB has the opportunity to completely or partially block LTN while blocking the anterior branches of intercostal nerves. The results of our study also suggest that SSPB has a clear advantage of improving VAS scores as compared to DSPB. This advantage may be partly resulted from the long thoracic nerve laying on the surface of the serratus anterior muscle. Furthermore, because of their origin and trajectory, they cannot be blocked by DSPB. Moreover, SSPB is also safer than DSPB because DSPB has higher risk of harming the pleura.

In addition, there was no significant difference in the PONV incidence among 3 groups. However, we also observed a slightly higher incidence of PONV in patients receiving SSPB. Although not statistically significant, we will further explore this phenomenon and eliminate interference factors, such as the use of prophylactic antiemetics and prolonged fasting, in future studies.

The pain mechanism after thoracic surgery is complicated, involving somatic movement and autonomic nervous system.^[25]^ In this study, the advantage of combination of patient-controlled intravenous analgesia and SSPB was initially reflected as an ideal analgesic solution after VATS. However, when scar is present, such as the case when the latissimus dorsi muscle is dissected off from the serratus anterior muscle fascial plane during axillary lymph node dissection, adequate local anesthetic spread may not occur using the SSPB technique.^[14]^ Since DSPB can provide long-lasting pain relief in otherwise difficult clinical scenarios, we recommend DSPB as a useful supplement. The correct DSPB is to inject local anesthetics over the ribs. Subsequently the medication immediately diffuses at a linear pattern, and further separates the anterior serratus and intercostal external muscles. It must be noted that, during operation, if the local anesthetics is mistakenly injected into the muscle, medication will yield a linear diffusion as well. But meanwhile it will radiate to the venter of the serratus anterior muscle. During the entire injection process, it is necessary to pay close attention to the bevel of the needle, and pay special attention to the diffusion of the local anesthetics during the injection to avoid intramuscular injection into the anterior serratus muscle.

There were some limitations in this study, firstly, we did not evaluate the postoperative analgesic effect of patients with VAS during cough or exercise because of the following reason: pain is the subjective and integrated feeling of the patient. Whether the discomfort is caused by pain during rest or cough, the VAS score can respond uniformly. Secondly, unfortunately, at the time of our project implementation, volume used on incentive spirometry was not included in the analysis due to the loss of some data. Thirdly, Biswas et al^[26]^ proposed in the study of “*A Cadaveric and Clinical Evaluation*” that the high-capacity double injection technology can widely and uniformly diffuse the dye in the anterior chest wall and axillary regardless of the injection surface of SAM. However, in this experiment, the optimal concentration and volume of local anesthetic drugs were not involved. Moreover, after the confirmation on the benefits of SSPB within 24 hours, we will study the continuous infusion through a catheter to maintain an effective postoperative analgesia over several days.

## 5 Conclusion

In summary, we observed that the serratus plane block can provide adequate postoperative analgesia for patients undergoing thoracoscopy lobectomy, and that over time, SSPB exhibited more stable and longer-lasting effect than DSPB did. Therefore, when considering a single-injection serratus plane block for the postoperative analgesia of thoracoscopy patients, we preferred to recommend SSPB more frequently. The results of this research can provide reference for multimodal analgesia after thoracoscopy surgery and a more detailed perioperative analgesia strategy in the clinic.

## Author contributions

Jianping Yang and Lan Qiu designed the study and Lan Qiu drafted the manuscript. Xiaoxuan Bu and Min Li collected the patient data. Lan Qiu, and Jiang Shen performed the statistical analysis. Linyi Yang, Qingrong Xu and Yongjun Chen contributed to manuscript review. All authors have read and approved the final manuscript.

**Conceptualization:** Lan Qiu, Jianping Yang.

**Data curation:** Lan Qiu, Xiaoxuan Bu, Jiang Shen, Min Li.

**Formal analysis:** Lan Qiu, Xiaoxuan Bu, Jiang Shen, Linyi Yang, Qingrong Xu, Jianping Yang.

**Funding acquisition:** Jianping Yang.

**Investigation:** Lan Qiu, Xiaoxuan Bu, Jianping Yang.

**Methodology:** Min Li.

**Project administration:** Jianping Yang.

**Resources:** Lan Qiu, Linyi Yang, Yongjun Chen.

**Software:** Lan Qiu, Xiaoxuan Bu, Jiang Shen, Min Li, Linyi Yang, Qingrong Xu.

**Supervision:** Jiang Shen, Qingrong Xu.

**Validation:** Lan Qiu, Xiaoxuan Bu, Jianping Yang.

**Writing – original draft:** Lan Qiu.

**Writing – review & editing:** Lan Qiu, Jianping Yang.
